# Dectin-3 Is Not Required for Protection against *Cryptococcus neoformans* Infection

**DOI:** 10.1371/journal.pone.0169347

**Published:** 2017-01-20

**Authors:** Althea Campuzano, Natalia Castro-Lopez, Karen L. Wozniak, Chrissy M. Leopold Wager, Floyd L. Wormley

**Affiliations:** 1 Department of Biology, The University of Texas at San Antonio, San Antonio, TX, United States of America; 2 South Texas Center for Emerging Infectious Diseases, The University of Texas at San Antonio, San Antonio, TX, United States of America; University of Minnesota, UNITED STATES

## Abstract

C-type lectin receptors (CLRs) are diverse, trans-membrane proteins that function as pattern recognition receptors (PRRs) which are necessary for orchestrating immune responses against pathogens. CLRs have been shown to play a major role in recognition and protection against fungal pathogens. Dectin-3 (also known as MCL, Clecsf8, or Clec4d) is a myeloid cell-specific CLR that recognizes mycobacterial trehalose 6,6’-dimycolate (TDM) as well as α-mannans present in the cell wall of fungal pathogens. To date, a potential role for Dectin-3 in the mediation of protective immune responses against *C*. *neoformans* has yet to be determined. Consequently, we evaluated the impact of Dectin-3 deficiency on the development of protective immune responses against *C*. *neoformans* using an experimental murine model of pulmonary cryptococcosis. Dectin-3 deficiency did not lead to increased susceptibility of mice to experimental pulmonary *C*. *neoformans* infection. Also, no significant differences in pulmonary leukocyte recruitment and cytokine production were observed in Dectin-3 deficient mice compared to wild type infected mice. In addition, we observed no differences in uptake and anti-cryptococcal activity of Dectin-3 deficient dendritic cells and macrophages. Altogether, our studies show that Dectin-3 is dispensable for mediating protective immune responses against pulmonary *C*. *neoformans* infection.

## Introduction

*Cryptococcus neoformans*, the predominant etiological agent of cryptococcosis, is an opportunistic fungal pathogen that is responsible for approximately one million cases of cryptococcal meningitis globally, resulting in over 620,000 deaths each year [1]. *C*. *neoformans* predominantly affects immunocompromised individuals, particularly patients with suppressed T cell-mediated immunity (CMI), including AIDS patients and solid organ transplant recipients [2–5]. Currently, there are no anti-fungal vaccines approved for human use, and at least one third of patients suffering from cryptococcal meningitis will undergo clinical and/or mycological failure [6, 7]. It is therefore imperative to develop new anti-fungal drugs, vaccines and/or immune-based therapies that elicit protection against cryptococcosis.

*C*. *neoformans* is found ubiquitously in the environment, and exposure via inhalation of yeast or desiccated basidiospores into the lungs occurs as early as infancy [8]. Since inhalation is the primary route of infection, identification and eradication by resident phagocytes, namely dendritic cells (DCs) and alveolar macrophages (Mϕs), is essential [9–12]. Innate immune cells are able to survey their environment and recognize pathogens via an arsenal of highly-conserved pattern recognition receptors (PRRs). PRRs recognize pathogen associated molecular patterns (PAMPs) such as branched mannans, β-glucans and chitins associated with the fungal cell wall [13, 14]. C-type lectin receptors (CLRs) are PRRs found on myeloid cells such as Mϕs and DCs that can recognize carbohydrate moieties present on fungal pathogens. Key CLR members demonstrated to participate in fungal immunity include: Dectin-1, Dectin-2, Mincle, and more recently, Dectin-3 [14–21]. CLR binding to its respective ligand triggers the activation of multiple signaling cascades through the recruitment of coupled- phosphorylated tyrosine residues of the spleen tyrosine kinase (Syk)/Caspase recruitment domain 9 (CARD9)/NF-kB-dependent signaling pathway that results in augmented reactive oxygen species production, increased phagocytosis, and the release of pro-inflammatory cytokines that can enhance protective immune responses [22–24]. Dectin-3 (also known as Clec4D, Clecsf8, MCL), which contains a short cytoplasmic tail without a signaling motif, shares the critical adaptor molecule, CARD9, that is critical during protection against cryptococcal infections [25] and is necessary for anti-fungal immunity in humans [26]. The role of Dectin-3 in promoting antifungal immunity against *Candida spp*., *Fonsecaea pedrosoi* and *Blastomyces dermatitidis* infections have been investigated [21, 27–29]. Consequently, we endeavored to determine the role of Dectin-3 in mediating protection against pulmonary *C*. *neoformans* infection using an experimental murine model of pulmonary cryptococcosis. Our studies show that Dectin-3 deficiency does not have a significant impact on mortality and phagocyte anti-*Cryptococcus* activity in mice given an experimental pulmonary *C*. *neoformans* infection. Thus, Dectin-3 is not universally required to mediate antifungal immunity.

## Materials and Methods

### Ethics Statement

All animal experiments were conducted following NIH guidelines for housing and care of laboratory animals and in accordance with protocols approved by the Institutional Animal Care and Use Committee (protocol number MU021) of the University of Texas at San Antonio. A scoring-system for assessment of animal distress was established before infection experiments were started. On the basis of these guidelines general condition and behavior of the animals was controlled by well-educated and trained staff. Depending on the progress of the disease, animals were monitored twice daily during the “day-phase” (7:00 am to 7:00 pm). In order not to disturb the circadian rhythm of the animals, there was no monitoring after 7:00 pm. Humane endpoint by CO_2_ asphyxiation followed by cervical dislocation was conducted if death of the animals during the following hours was to be expected.

### Mice

Male and female Dectin-3 KO (or Clecsf8^-/-^, MCL, CLEC4d KO) mice on a C57BL/6 background and their appropriate control mice (C57BL/6) were a generous gift from Dr. Marcel Wüthrich (University of Wisconsin–Madison, Madison, WI). All animal experiments were approved by The University of Texas at San Antonio Institutional Animal Care and Use Committee (IACUC) and mice were handled according to IACUC guidelines.

### Strains and Media

*Cryptococcus neoformans* strains H99 (serotype A, mating type α), mCherry producing mutant, KN99mCH (serotype A, KN99 mating type α), a kind gift from Dr. Jenny Lodge (Washington University, St. Louis, MO) and *C*. *neoformans* strain 52D (serotype D) were recovered from 15% glycerol stocks stored at -80°C and maintained on yeast peptone dextrose (YPD) media agar plates (Becton Dickinson, Sparks, MD). Yeast cells were grown for 16–18 h at 30°C with shaking in liquid YPD broth, collected by centrifugation, washed three times with sterile phosphate buffered saline (PBS), and viable yeasts were quantified using trypan blue dye exclusion on a hemacytometer.

### Pulmonary Cryptococcal Infections and Fungal Burden

Mice were anesthetized with 2% isoflurane utilizing a rodent anesthesia device (Eagle Eye Anesthesia, Jacksonville, FL) and were infected via the intranasal route with 1 x 10^4^ colony forming units (CFUs) of *C*. *neoformans* strain H99 or 52D in 50 μl of sterile PBS. The inocula used for the nasal inhalation were verified by quantitative culture on YPD agar. Mice were euthanized on predetermined days by CO_2_ inhalation followed by cervical dislocation and lung tissues excised. The left lobe of the lung was removed and homogenized in 1 ml of sterile PBS as previously described [30] followed by culture of 10-fold dilutions of each homogenate on YPD agar supplemented with chloramphenicol. CFUs were enumerated following incubation at 30°C for 48 h. For survival studies, mice were inoculated as stated above, monitored twice daily and sacrificed if moribund.

### Pulmonary Leukocyte Isolation

Lungs of WT and Dectin-3 KO mice (n = 5/group) were excised on days 7 and 14 post-inoculation as previously described [30]. Lungs were then digested enzymatically at 37°C for 30 min in 10 ml digestion buffer (RPMI 1640 and 1 mg/ml collagenase type IV [Sigma-Aldrich, St. Louis, MO]) with intermittent (every 10 min) stomacher homogenizations. The digested tissues were then successively filtered through sterile 70- and 40-μm nylon filters (BD Biosciences, San Diego, CA) to enrich for leukocytes and the cells then washed three times with sterile Hank's Balanced Salt Solution (HBSS). Erythrocytes were lysed by incubation in NH_4_Cl buffer (0.859% NH_4_Cl, 0.1% KHCO_3_, 0.0372% Na_2_EDTA [pH 7.4]; Sigma-Aldrich) for 3 min on ice followed by a 2-fold excess of sterile PBS. T cells were first depleted by α-CD3 antibodies and subsequent binding using anti-biotin magnetic beads (Miltenyi Biotec, Auburn, CA). The leukocyte population was then enriched for Mϕs using biotinylated α-F4/80 antibodies and subsequent binding using anti-biotin magnetic beads (Miltenyi Biotec) according to manufacturer’s recommendations. Following isolation of F4/80^+^ Mϕs, the remaining cells were enriched for dendritic cells (DCs) via positive selection by labeling cells with α-CD11c labeled magnetic beads (Miltenyi Biotec) according to manufacturer’s recommendations. Purity of each cell type was validated via flow cytometry using labeling check (Miltenyi Biotec) with F4/80^+^ and CD45 antibodies (eBioscience) for Mϕs and CD11c^+^ and CD11b^+^ antibodies for dendritic cells (purity of Mϕs and DCs was routinely >85% and >95%, respectively).

### Flow Cytometry

Standard methodology was employed for the direct immunofluorescence of pulmonary leukocytes. Briefly, in 96-well U-Bottom plates containing 1 x 10^6^ cells in 100 μl of PBS plus 2% FBS (FACS buffer) were incubated with 100 μl of Fc block (BD Bioscience) diluted in FACS buffer to prevent nonspecific binding of antibodies to cellular Fc receptors. Subsequently, an optimal concentration of fluorochrome-conjugated antibodies (between 0.06 and 0.5 μg per 1 x 10^6^ cells) was added and cells incubated for 30 min at 4°C. Following incubation, the cells were washed three times with FACS buffer and fixed in 200 μl of 2% ultrapure formaldehyde (Polysciences, Inc., Warrington, PA) diluted in FACS buffer (fixation buffer). Cells incubated with either FACS buffer alone or single fluorochrome-conjugated antibodies were used to determine positive staining and spillover/compensation calculations and the flow cytometer determined background fluorescence. The samples were analyzed using BD FACSArray software on a BD FACSArray flow cytometer (BD Biosciences). Dead cells were excluded on the basis of forward angle and 90° light scatter. For data analyses, 30,000 events (cells) were evaluated from a predominantly leukocyte population identified by back-gating from CD45^+^-stained cells. The absolute number of leukocytes (CD45^+^ cells), CD4^+^/CD3^+^ T cells, CD8^+^/CD3^+^ T cells, CD19/CD45^+^ B cells, 1A8^+^/CD45^+^ polymorphonuclear leukocytes (PMNs), F4/80^+^/CD45^+^ Mϕs, CD11c^+^/CD11b^+^ DCs, SiglecF^+^/CD11b^int^ eosinophils, B220^+^/CD11c^+^/PDCA-1^+^ plasmacytoid DCs (pDCs), γδ^+^/CD45^+^ γδ T cells, NKp46^+^/DX5^+^ NK cells, and CD4^+^/NKp46^+^/DX5^+^ NKT cells was determined by multiplying the percentage of each gated population by the total number of CD45^+^ cells.

### Cytokine Analysis

Cytokine levels within lung tissue homogenates were analyzed using the Bio-Plex protein array system (Luminex-based technology, Bio-Rad Laboratories, Hercules, CA). Briefly, lung tissue was excised and homogenized in ice-cold sterile PBS (1 ml). An aliquot (50 μl) was taken to quantify the pulmonary fungal burden and an anti-protease buffer solution (1 ml) containing PBS, protease inhibitors (inhibiting cysteine, serine, and other metalloproteinases) and 0.05% Triton X-100 was added to the homogenate. Samples were then clarified by centrifugation (3500 rpm) for 10 minutes. Supernatants from pulmonary homogenates were assayed for the presence of interleukin (IL)-2, IL-4, IL-5, IL-10, IL-12(p70), IFN-γ, tumor necrosis factor (TNF)-α, and granulocyte-macrophage colony stimulating factor (GM-CSF) according to the manufacturer’s instructions.

### Isolation of Bone Marrow Derived Dendritic Cells (BMDC) and Macrophages (BMM)

BMDC culture was performed as previously described [31, 32]. Briefly, bone marrow was flushed from femurs and tibiae of mice. Cells were then washed, counted and plated at a concentration of 2 x 10^5^ cells/ml in RPMI complete medium (RPMI 1640 supplemented with 10% heat-inactivated fetal bovine serum, 2 mM L-glutamine, 100 U penicillin/ml, 100 μg of streptomycin/ml, and 50 mM 2-mercaptoethanol) supplemented with 20ng/ml of recombinant murine GM-CSF (Peprotech, Rocky Hill, NJ) for DCs and 4 x 10^5^ cells/ml supplemented with 20ng/ml of murine M-CSF (Peprotech) for Mϕs. Cells were incubated at 37°C and 5% CO_2_, half the medium replaced every three days, and cells harvested on day 8. The leukocyte population was then enriched for DCs by first depleting Mϕs using biotinylated α-F4/80^+^ antibody (eBioscience) followed by incubation with anti-biotin conjugated magnetic beads (Miltenyi Biotec). The flow-through was then enriched for DCs by positive selection using α-CD11c magnetic beads (Miltenyi Biotec). Enrichment of Mϕs was accomplished by gently washing Mϕs off the bottom of the petri dish plate using Versene (Gibco) and removing DCs using α-CD11c magnetic beads (Miltenyi Biotec). The flow-through was then enriched for Mϕs using biotinylated α-F4/80 antibody followed by incubation with anti-biotin conjugated magnetic beads (Miltenyi Biotec). Purity for enriched BMDC as well as BMM population was routinely >95%.

### Uptake and Killing Assays

Pulmonary DCs and Mϕs were isolated as described above [31]. Cells were counted and plated at a concentration of 1 x 10^6^ cells and incubated with an mCherry expressing strain of *Cryptococcus*, KN99mCH, at either a 10:1 or 20:1 ratio for 6 hours with murine antibody against *Cryptococ*cus GXM (a kind gift from Thomas Kozel, University of Nevada, Reno NV). Mϕs were labeled with PE-F4/80 and APC-CD45 antibodies (eBioscience), while DCs were labeled with PE-CD11c and APC-CD11b (eBioscience) for 30 mins at 4°C, washed with FACS buffer and then cells were fixed in 2% ultra-pure formaldehyde. Analysis was done in triplicate and quantified using ImageStreamX IDEAS® 6.1 software (Millipore) after 100,000 cells were collected. For killing assays, enriched pulmonary, and bone marrow-derived DCs and Mϕs were incubated with *C*. *neoformans* strain H99 for 24 hours in opsonizing conditions (using 1ug/ml anti-GXM antibody) in complete medium at 37°C and 5% CO_2_. At predetermined time points, murine cells were lysed for 15 minutes with sterile water, and then 1:10 dilutions were plated onto YPD agar and CFUs enumerated as previously described in [30]. Percent inhibition was calculated by dividing the CFUs of H99 cultured with DCs or Mϕs by CFUs of H99 alone then then multiplying the result by 100.

### Statistical Analysis

Survival data were analyzed using log-rank test to detect statistically significant differences using GraphPad Prism Version 6.0 for Macintosh (GraphPad Software, San Diego, CA). The Mann-Whitney test was used to analyze fungal burden and one-way analysis of variance (ANOVA) with Tukey’s post-test was used for Mϕ and DC uptake and killing assays in order to detect significant differences (GraphPad Software). Significant differences were defined as *P* < 0.05 (*).

## Results

### Dectin-3 is Not Essential for Protection against Pulmonary Infection with *C*. *neoformans* in Mice

**Fig 1:**
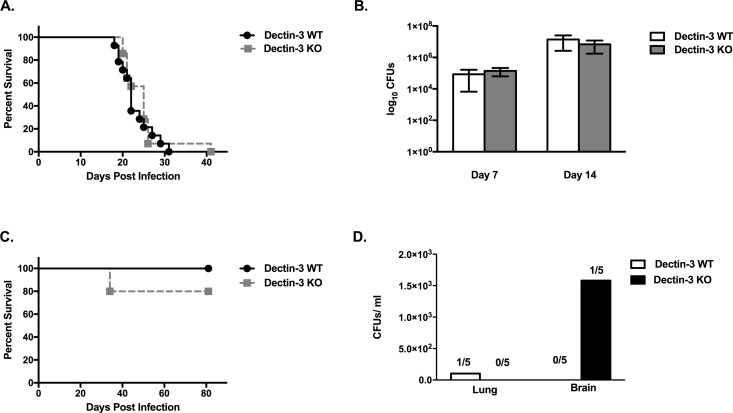
Dectin-3 is not necessary for survival or control of pulmonary fungal burden following infection with *C*. *neoformans*. C57BL/6 (WT) and Dectin-3 KO mice were given an intranasal inoculation with *C*. *neoformans* strain H99 (serotype A). Mice were observed up to Day 41 for survival (A) and pulmonary fungal burden (B) was analyzed at days 7 and 14 post-inoculation. Additionally, C57BL/6 (WT) and Dectin-3 KO mice were given an intranasal inoculation with *Cryptococcus* strain 52D (serotype D). Mice were observed up to Day 80 for survival (C). Survival data shown are representative of one study using 15 mice per group. Fungal burden data shown are mean ± SEM from three independent experiments performed using 5 mice per group per time point.

Previous studies to determine the immunological function of the pattern recognition receptor Dectin-3 have demonstrated its key significance in protection against mycobacterial infections [33]. Additionally, a role for Dectin-3 in mediating protection against *Candida albicans* infection has been demonstrated [27]. However, a role for Dectin-3 during the protective immune response to pulmonary *C*. *neoformans* infection is yet to be determined. Consequently, we investigated the overall impact of Dectin-3 deficiency during an experimental pulmonary *C*. *neoformans* infection in mice. Dectin-3 KO mice and WT controls received an intranasal inoculation with *C*. *neoformans* strain H99. Survival (morbidity) was monitored for greater than 40 days post-inoculation, while pulmonary fungal burden was evaluated in a separate group of infected mice at select time points post-inoculation (Fig 1A and 1B). WT and Dectin-3 KO mice demonstrated an equivalent susceptibility to pulmonary *C*. *neoformans* H99 infection (median survival of 22 and 25 days post infection for WT and Dectin-3 KO mice, respectively; Fig 1A). Also, no significant differences were observed in the pulmonary fungal burdens of Dectin-3 KO mice compared to WT mice on days 7 and 14 post inoculation with *C*. *neoformans* strain H99 (Fig 1B). Additionally, we determined the impact of Dectin-3 deficiency on the overall protection against experimental pulmonary cryptococcosis using a less virulent strain of *Cryptococcus*, 52D. Dectin-3 KO mice and WT mice received an intranasal inoculation with *C*. *neoformans* strain 52D, and their survival was monitored for 80 days post-inoculation (Fig 1C). Experimental pulmonary infection with *C*. *neoformans* strain 52D typically results in a chronic pulmonary infection in C57BL/6 mice [34, 35]. All WT mice infected with *C*. *neoformans* strain 52D were alive and appeared healthy upon termination of the experiment at day 80 post-inoculation. Dectin-3 KO mice exhibited an 80% survival rate upon termination of the study. Postmortem analysis suggested that WT and Dectin-3 KO mice resolved the pulmonary infection as only one WT infected mouse was observed to contain yeast in their lungs (Fig 1D). Also, little evidence of CNS infection was observed as yeast was observed in the brain of only one Dectin-3 KO mouse (Fig 1D). These results indicate that Dectin-3 is not required for survival against pulmonary *C*. *neoformans* infection.

### Dectin-3 Deficiency Does Not Alter Pulmonary Leukocyte Infiltration during *C*. *neoformans* Pulmonary Infection

**Fig 2:**
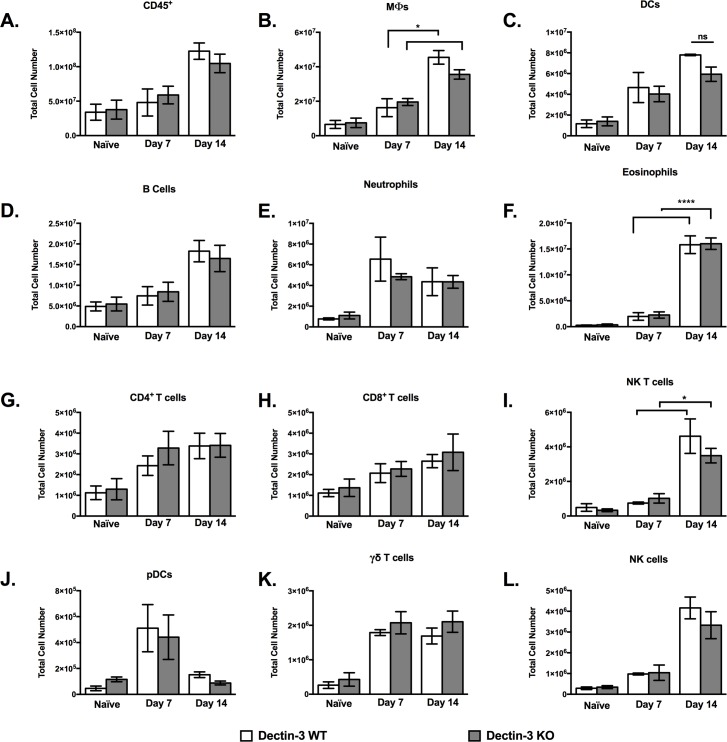
Dectin-3 deficiency does not impact pulmonary leukocyte recruitment during immune response to *C*. *neoformans* infection. C57BL/6 (WT) and Dectin-3 KO mice were infected via intranasal inoculation with *C*. *neoformans* strain H99. Lungs were excised at days 7 and 14 post-inoculation and pulmonary infiltrates analyzed by flow cytometry. Leukocytes were labeled with anti-CD45 antibodies for total leukocytes (A) or dual labeled with anti-CD45 and antibodies specific for cell type (B-L) and were analyzed by flow cytometry. Data shown are the mean ± of SEM absolute cell numbers from three independent experiments performed using 5 mice per group per time point per experiment. Significant differences were defined as *P* < 0.05 (*), *P* < 0.01 (**) *P* < 0.001 (***), *P* < 0.0001(****).

We observed no defect in overall survival and ability to control a pulmonary *C*. *neoformans* infection in Dectin-3 deficient mice compared to WT infected mice. Next, we evaluated whether Dectin-3 deficiency results in an altered leukocyte profile during a pulmonary *C*. *neoformans* infection. To quantify pulmonary recruitment during infection, flow cytometry analysis of pulmonary leukocytes isolated from enzymatically dispersed lungs of WT and Dectin-3 KO mice was performed at days 7 and 14 post-inoculation. Naïve mice were used as a baseline to compare leukocyte populations (Fig 2A–2L). An overall increase in total leukocyte infiltration to the lungs of both WT and KO infected mice was observed on D14 post-inoculation compared to naïve counterparts. However, we observed no statistically significant differences in total CD45^+^ leukocyte infiltration between WT and Dectin-3 KO mice at either time point (Fig 2A). In addition, there were no significant differences in all infiltrating pulmonary leukocyte subsets tested including: Mϕs, DCs, B cells, neutrophils, eosinophils, CD4^+^ T cells, CD8^+^ T cells, NK T cells, pDCs, γδ T cells, and NK cells between WT and KO mice on both day 7 and 14 post-inoculation (Fig 2B–2L). We did observe an increase of Mϕs (*p* < 0.05), NK T cells (*p* < 0.05), and eosinophils (*p* < 0.00001) in both infected WT and Dectin-3 KO mice on day 14 compared to day 7 post inoculation. Altogether, these data suggest that Dectin-3 deficiency does not significantly alter the phenotype of the cellular immune response to experimental pulmonary cryptococcosis.

### Dectin-3 Deficiency Does Not Alter Pulmonary Cytokine Profile during Pulmonary *C*. *neoformans* Infection

**Fig 3:**
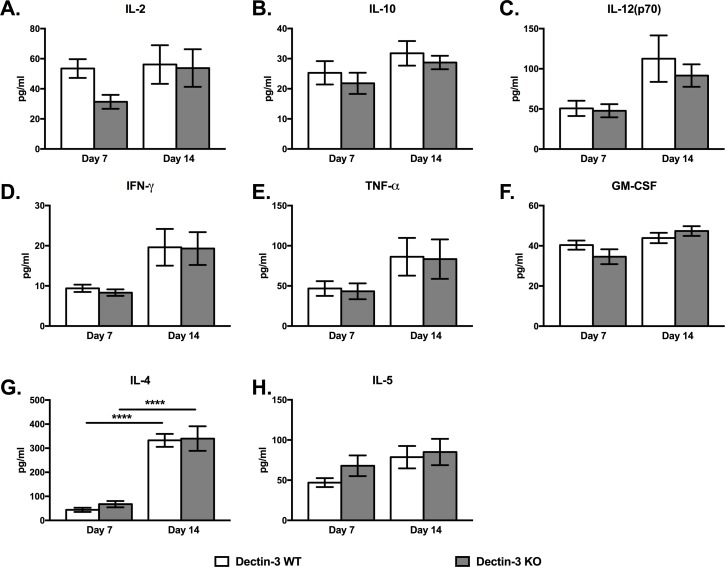
Phenotype of cytokine response is similar in WT and Dectin-3 deficient mice during pulmonary infection with *C*. *neoformans*. WT mice and Dectin-3 KO mice were given a pulmonary infection with *C*. *neoformans* strain H99. Lungs were excised at day 7 and 14 post-inoculation and the right lobe homogenized and cytokine levels analyzed. Data represent the mean ± SEM from 2 independent experiments performed using 5 mice per group per time point.

As a result of observing a lack of distinction between survival and leukocyte infiltration, we determined the putative impact of Dectin-3 deficiency on cytokine production during a pulmonary infection with *C*. *neoformans* strain H99. Lung homogenates were prepared from WT and Dectin-3 KO mice on days 7 and 14 post inoculation and analyzed for Th1-type (IL-2, IL-12p70, IFN-γ,) Th2-type (IL-4, IL-5, and IL-10), GM-CSF and TNF-α protein levels (Fig 3). Overall, cytokine production between Dectin-3 KO and WT mice was not significantly different during the time course evaluated. No significant differences in IL-2, IL-4, IL-5, IL-10, IL-12p70, IFN-γ, GM-CSF, or TNF-α levels were observed between the two groups of mice on day 7 or 14 post inoculation (Fig 3A–3H). Levels of IL-4 appeared to be significantly increased in the lungs of both WT and Dectin-3 KO mice on day 14, compared to day 7, post inoculation (*p* < 0.0001) which is suggestive of an overall trend toward a Th2-type response that is associated with progressive cryptococcal disease.

### Dendritic Cell and Macrophage Phagocytic and Anti-Cryptococcal Activities are Not Impaired Due to Dectin-3 Deficiency

**Fig 4:**
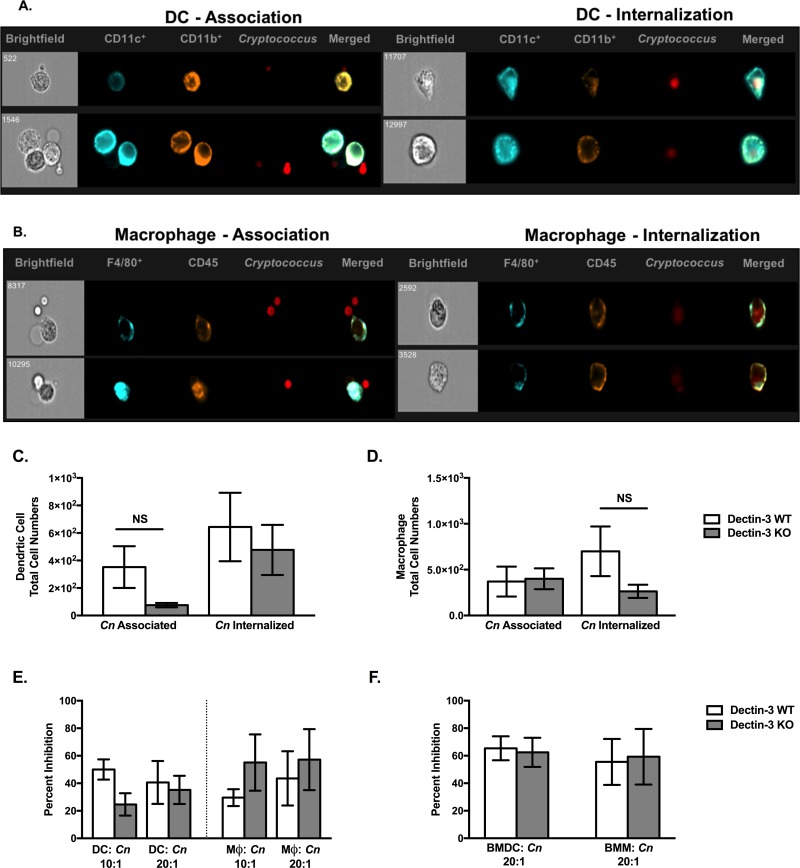
Phagocytic and anti-cryptococcal activity of pulmonary and bone marrow derived DCs and macrophages *in vitro* are not impacted by Dectin-3 deficiency. Pulmonary DCs (Fig 4A) and Mϕs (Fig 4B) were islolated from WT and Dectin-3 KO mice and then incubated with mCherry expressing *C*. *neoformans* strain KN99mCH (KN99 mating type α) at a 10:1 ratio for 6 hr (Fig 4C and Fig 4D). Cells were analyzed at 40x magnification for uptake and association by the ImageStreamX-Imaging Flow Cytometer-ISX-MKII. Anti-cryptococcal activity of naïve pulmonary DCs and Mϕs was measured following culture of the cells with *C*. *neoformans* strain H99 for 24h at the designated ratios (Fig 4E). The cells were subsequently lysed and CFUs determined. Bone marrow derived DC and Mϕ anti-*Cryptococcus* activity were also analyzed following 24h incubation with *C*. *neoformans* strain H99 (Fig 4F).

Mϕs and DCs are among the first line of defense following inhalation of *Cryptococcus*. Thus, we sought to determine the overall impact of Dectin-3 deficiency on the phagocytic and anti-cryptococcal activity of Mϕs and DCs. To do this, DCs and Mϕs were isolated from the lungs of naïve Dectin-3 KO and WT mice. The DCs and Mϕs were incubated with an m-Cherry expressing *C*. *neoformans* strain, KN99mCH, in order to evaluate its uptake/association at 6h post incubation using an ImageStreamX-Imaging Flow Cytometer ISX-MKII and accompanying ImageStreamX IDEAS® 6.1 software. We observed no significant differences in the number of cryptococci associated with or internalized by DCs (Fig 4A and 4C) or pulmonary Mϕs (Fig 4B and 4D) of WT compared to Dectin-3 KO mice. In addition to uptake analysis, the anti-cryptococcal activity of DCs and Mϕs from Dectin-3 deficient mice were also determined in comparison to cells from WT mice. The DCs and Mϕs were incubated with *C*. *neoformans* strain H99 at effector to target ratios of 10:1 and 20:1 for 24h. The cells were then lysed to release any intracellular cryptococci and the fungal burden determined to quantify the antifungal efficiency of both DCs and Mϕs (Fig 4E). We observed no significant difference in the percent inhibition of *Cryptococcus* by DCs or Mϕs from WT compared to Dectin-3 KO mice. Additionally, we sought to evaluate the anti-cryptococcal capabilities of bone marrow derived DCs (BMDCs) and bone marrow derived Mϕs (BMMs) (Fig 4F). Although there appeared to be an increase in percent inhibition by BMDCs and BMMs compared to primary derived pulmonary DCs and Mϕs, there were no significant differences in cryptococcal inhibition between cells derived from WT and Dectin-3 KO mice. Altogether, these studies demonstrate that Dectin-3 is dispensable for the anti-microbial activity of Mϕs and DCs against *C*. *neoformans* infection.

## Discussion

Germline encoded PRRs recognize a variety of microbial moieties which, once engaged, result in the activation of anti-microbial host defense and stimulation of adaptive immune responses. The role of CLRs during cryptococcosis are of interest as recent studies have defined their role in the recognition of carbohydrate moieties and host defense against other fungal pathogens [21, 27–29]. Particularly, Dectin-3 has been shown to promote antifungal immunity against *Candida spp*., *Fonsecaea pedrosoi* and *Blastomyces dermatitidis* infections [21, 27–29]. Nonetheless, our studies clearly show that Dectin-3 deficiency has no deleterious impact on the phenotype of the host immune response against *C*. *neoformans*.

Earlier studies demonstrated the importance of mannose receptor (MR or CD206) and dendritic-cell-specific ICAM-3 grabbing nonintegrin (DC-SIGN or CD209) in recognition of heavily mannosylated cryptococcal mannoproteins [36, 37]. Mice deficient in these MRs were more susceptible to experimental *C*. *neoformans* infection compared to WT mice. Dectin-2 is postulated to oppose Th2-type responses and IL-4-dependent mucin production in the lungs following infection with a serotype D strain of *C*. *neoformans* [38]. However, Dectin-2 deficiency did not impact the production of Th1-type or Th17-type cytokines, and proinflammatory cytokines or clearance of the serotype D strain. The impact of Dectin-2 deficiency could possibly vary depending on the *Cryptococcus* serotype or strain studied. Nonetheless, we observed no difference in the capacity of Dectin-3 KO mice to resolve experimental pulmonary infections with serotype A or serotype D strains of *Cryptococcus*. Thus, our results do not appear to be *Cryptococcus* strain specific. Our observations are more in line with previous studies demonstrating that Dectin-1, which recognizes β-glucans in a variety of fungi including *C*. *albicans* [39], is not required for protection against *Cryptococcus* [40]. Thus, Dectin-1 and Dectin-3, which are clearly important during the host defense to some fungal pathogens, are not universally required for anti-fungal immunity.

*C*. *neoformans* possesses the ability to shield its cell wall components behind its large polysaccharide capsule [41]. The capsule is primarily composed of high-molecular weight polysaccharides glucuronoxylomannan (GXM) and galactoxylomannan (GalXM) and mannoproteins anchored to the cell wall [42]. The cell wall itself is composed of chitin, chitosan, glucans and glycoproteins. Thus, carbohydrates present in the yeast’s cell wall are ideal PAMPs for recognition by the Dectin-3 receptor present on phagocytic cells. However, the *Cryptococcus* capsule could shield potential Dectin-3 ligands thus allowing the yeast to evade potential stimulation of host responses that may occur following engagement of the Dectin-3 receptor.

Host responses that are triggered following the recognition fungal PAMPs by PRRs is amplified by cooperation between Dectin-3 and other CLR members. Dectin-3 is capable of forming heterodimeric complexes with Mincle receptor following stimulation of TDM [43]. Also, signaling through adaptor molecule MyD88-associated with toll-like receptors can also drive surface expression of Dectin-3 and Mincle following microbial stimulation [44]. Studies showed that Dectin-3 deficient mice were more susceptible to *Candida* infections and formation of Dectin-3/Dectin-2 heterodimer complexes had a greater affinity to recognize α-mannans on the hyphae of *C*. *albicans* [27]. However, Dectin-3 deficiency does not appear to alter the phenotype of the immune response against pulmonary *C*. *neoformans* infection and any role for Dectin-3 in the amplification of other CLR activities against *Cryptococcus* is not apparent in our studies. Nonetheless, we can’t rule out the possibility that redundancy in the ability of other PRRs to compensate for Dectin-3 deficiency may mask any possible function of Dectin-3 during the anti-*Cryptococcus* immune response.

In conclusion, we have extensively characterized the role of the Dectin-3 receptor during pulmonary cryptococcosis using an experimental murine model of pulmonary *C neoformans* infection. Dectin-3 deficient mice did not display any differences in survival, fungal burden, pulmonary cytokine production, or lung leukocyte recruitment during *C*. *neoformans* infection compared to that observed in WT infected mice. Additionally, we showed that Dectin-3 deficiency had no impact on the anti-microbial activity of Mϕs or DCs against *C*. *neoformans*. Altogether, these studies indicate that Dectin-3 is not required for recognition and anti-fungal host responses against *C*. *neoformans* and is not universally required for anti-fungal immunity.

## Abbreviations

CLRs: C-type lectin receptors
Mϕ: macrophages
DCs: dendritic cells
PRRs: pattern recognition receptors
PAMPs: Pathogen Associated Molecular Patterns
Dectin: DC associated C-type lectin
MCL: Macrophage C-type lectin
